# UrdA Controls Secondary Metabolite Production and the Balance between Asexual and Sexual Development in *Aspergillus nidulans*

**DOI:** 10.3390/genes9120570

**Published:** 2018-11-23

**Authors:** Sandesh S. Pandit, Jessica M. Lohmar, Shawana Ahmed, Oier Etxebeste, Eduardo A. Espeso, Ana M. Calvo

**Affiliations:** 1 Department of Biological Sciences, Northern Illinois University, 155 Castle Dr., Dekalb, IL 60115, USA; sha.sandesh@gmail.com (S.S.P.); jlohmar1@niu.edu (J.M.L.); ahmedshawana@gmail.com (S.A.); 2 Department of Applied Chemistry, Faculty of Chemistry, University of the Basque Country (UPV/EHU), Manuel de Lardizabal, 3, 20018 San Sebastian, Spain; oier.echeveste@ehu.eus; 3 Department of Cellular and Molecular Biology, Centro de Investigaciones Biológicas (C.S.I.C.), Ramiro de Maeztu 9, 28040 Madrid, Spain; eespeso@cib.csic.es

**Keywords:** *Aspergillus nidulans*, morphological development, secondary metabolism, central developmental pathway, sterigmatocystin, *brlA*, veA, epistasis, UrdA, transcription factor, mycotoxin

## Abstract

The genus *Aspergillus* includes important plant pathogens, opportunistic human pathogens and mycotoxigenic fungi. In these organisms, secondary metabolism and morphogenesis are subject to a complex genetic regulation. Here we functionally characterized *urdA*, a gene encoding a putative helix-loop-helix (HLH)-type regulator in the model fungus *Aspergillus nidulans*. *urdA* governs asexual and sexual development in strains with a wild-type *veA* background; absence of *urdA* resulted in severe morphological alterations, with a significant reduction of conidial production and an increase in cleistothecial formation, even in the presence of light, a repressor of sex. The positive effect of *urdA* on conidiation is mediated by the central developmental pathway (CDP). However, *brlA* overexpression was not sufficient to restore wild-type conidiation in the Δ*urdA* strain. Heterologous complementation of Δ*urdA* with the putative *Aspergillus flavus urdA* homolog also failed to rescue conidiation wild-type levels, indicating that both genes perform different functions, probably reflected by key sequence divergence. UrdA also represses sterigmatocystin (ST) toxin production in the presence of light by affecting the expression of *aflR*, the activator of the ST gene cluster. Furthermore, UrdA regulates the production of several unknown secondary metabolites, revealing a broader regulatory scope. Interestingly, UrdA affects the abundance and distribution of the VeA protein in hyphae, and our genetics studies indicated that *veA* appears epistatic to *urdA* regarding ST production. However, the distinct *fluffy* phenotype of the Δ*urdA*Δ*veA* double mutant suggests that both regulators conduct independent developmental roles. Overall, these results suggest that UrdA plays a pivotal role in the coordination of development and secondary metabolism in *A. nidulans*.

## 1. Introduction

Numerous fungal species synthesize a variety of natural products, also called secondary metabolites, which are not necessary for the organism to survive but can confer an ecological advantage [1,2,3]. Many fungal secondary metabolites produced are bioactive and have beneficial properties, whereas others are detrimental to plants, animals and human health. Among these harmful compounds are mycotoxins. Some of these mycotoxins are mutagenic, teratogenic and carcinogenic [4,5]. *Aspergillus nidulans*, a filamentous fungus that has been used as a model organism for more than 60 years [6], is capable of producing a mycotoxin known as sterigmatocystin (ST). Sterigmatocystin is similar to a highly carcinogenic compound known as aflatoxins (AF) [7,8,9,10]. Aflatoxins are produced by species phylogenetically related to *A. nidulans* such as *Aspergillus flavus*, *Aspergillus parasiticus*, and *Aspergillus nomius*. Both, ST and AF, are synthesized through a conserved metabolic pathway, where ST is the penultimate precursor of AF [11]. The genes involved in the production of ST and AF are clustered. The structural genes in these clusters are controlled by an endogenous regulatory gene called *aflR*, which encodes a transcription factor containing a binuclear zinc cluster domain [12,13,14,15]. *Aspergillus nidulans* also synthesizes other secondary metabolites, such as the beta-lactam antibiotic penicillin (PN) and the anti-tumoral secondary metabolite terrequinone A [16,17].

The genetic control of secondary metabolism and morphological development are connected [18,19]. *Aspergillus nidulans* develops sexually by producing fruiting bodies called cleistothecia where ascospores are formed. In addition, air-borne asexual spores are formed on specialized structures called conidiophores. Several genes involved in the regulation of asexual development have been identified and extensively characterized in *A. nidulans* [20,21,22,23]. Different exogenous and endogenous stimuli induce the expression of signaling genes, resulting in the modification of vegetative hyphae into asexual reproductive structures [24]. The genes involved in conidiophore formation are split into the upstream developmental activators (UDA) *flbA-E* and the central developmental pathway (CDP). The CDP consists of three genes known as *brlA*, *abaA* and *wetA*. They regulate the spatiotemporal expression of different genes involved in the formation of the conidiophore cell-types [20,23,25]. Absence or inactivation of *brlA*, the first gene in the CDP pathway, leads to aconidial colonies [26].

Etxebeste et al. [21] showed the importance of a transcription factor known as FlbB in the control of *brlA* expression. FlbB is detected at the tip of hyphae. Mutations in *flbB*, as well as other genes in the UDA signaling pathway, result in *fluffy* colonies (aconidial colonies with abundant aerial mycelium) [21,25]. *flbB* is not only required to induce asexual development but also to repress sexual development. Transcriptome analysis of the *flbB* deletion mutant revealed that the gene AN4394, called *urdA*, is positively regulated by *flbB* [27]. Oiartzabal-Arano et al. showed that disruption of *urdA* in *A. nidulans* reduces conidiation and the expression of *brlA*, while cleistothecial development was prematurely induced [27]. The same study showed that *urdA* deletion also resulted in an upregulation of two secondary metabolite genes of the *dba* gene cluster (AN7895/*cipB* and AN7898/*dbaD*), which is required for the synthesis of the antibiotic 2,4-dihydroxy-3-methyl-6-(2-oxopropyl) benzaldehyde or DHMBA [28]. 

Another well-known genetic link between morphogenesis and synthesis of natural products is VeA. This global regulator forms the VeA/VelB/LaeA heterotrimeric complex in *A. nidulans* and many other fungi [19]. In addition, VeA also interacts with light sensing proteins FphA and LreA-LreB [29]. The VeA protein is transported in a light-dependent manner to the nucleus by the α-importin KapA, which binds to the nuclear localization signal (NLS) of VeA [30,31] after the formation of a dimeric complex with VelB [32]. Inside the nucleus, VeA forms the heterotrimeric complex with VelB and LaeA, regulating secondary metabolite production and fungal development [19,33]. In light, cytoplasmic FphA negatively affects the transit of VeA to nuclei [29]. 

The role of UrdA in the control of development and secondary metabolism has only been initially characterized in the *veA1* mutant background. The *veA1* allele bears a mutation within the NLS of VeA, affecting the efficiency of translocation to the nucleus in the absence of light. This results in strains that conidiate in the absence of light. The goal of the current study is to characterize the role of UrdA in a wild-type *veA* background (*veA*^+^) in *A. nidulans*, particularly the investigation of its effects on asexual and sexual development as well as its possible role in the regulation of ST toxin production, PN biosynthesis and production of other secondary metabolites. Furthermore, we assessed whether *urdA* influences the abundance and subcellular localization of VeA, as well as the epistatic relationships between *urdA* and *veA*. We also evaluated its uniqueness or possible functional conservation with other fungal homologs by heterologously complementing the *urdA* deletion in *A. nidulans* with the homologous gene from the AF-producer and agriculturally important fungus *A. flavus*.

## 2. Materials and Methods

### 2.1. Sequence Search, Alignment and Phylogenetic Analyses

The deduced amino acid sequence of *A. nidulans urdA* (ANID_04394) was obtained from the Aspergillus Genome Database (Date accessed: 15 October 2017 [34]. The BLASTp search tool [35] was used to obtain putative orthologous sequences, the corresponding score and expected (e) values, sequence similarities and identities. Amino acid sequences were aligned using Clustal Omega [36] and alignments were visualized using Genedoc software. The phylogenetic tree was generated using Mega (version 4.0) and the Neighbor-joining method with a bootstrap value of 5000 [37]. Finally, UrdA orthologs of those *Aspergillus* species located in the same branch of the phylogenetic tree were again aligned for domain analysis. 

### 2.2. Fungal Strains and Culture Conditions

The *A. nidulans* strains used in this study are listed in Table S1. All the selected strains used for the characterization of *urdA* bear a *veA* wild-type allele. Strains were cultured on glucose minimal medium (GMM) [38], supplemented with the nutrients associated with the corresponding auxotrophic markers unless otherwise stated. For solid medium, agar (15 g/L) was added. Threonine minimal medium (TMM) [39], where threonine replaced glucose as the carbon source, was used to induce the *alcA* promoter. The strains were stored as 30% glycerol stocks at −80 °C. The experiments were carried out using three replicates. 

### 2.3. Generation of the *urdA* Deletion Strain (*∆urdA*)

The *A. nidulans ∆urdA* strain (TSSP1.1) was generated by transforming a 4.9 kb fusion PCR cassette constructed as previously described [40]. Specifically, first, 5′ and 3′ untranslated regions (UTRs) of *urdA* were PCR amplified from *A. nidulans* FGSC4 genomic DNA with primers urdA-P1 and urdA-P2 (all primers are listed in Table S2), and primers urdA-P3 and urdA-P4 respectively. The *pyrG* marker gene from *A. fumigatus* was PCR amplified from plasmid pFNO3 [30,41] using primers urdA-P5-pyrG and urdA-P6-pyrG. The 5′ UTR, 3′ UTR and *pyrG* fragments were fused using primers urdA-P7 and urdA-P8. Protoplasts of the host strain RJMP1.49 were transformed with the fusion-PCR cassette as previously described [40]. Transformants were confirmed by diagnostic PCR. 

Additionally, primers Anidpyro_5UTR_F and Anidpyro_3UTR_R were used to PCR amplify a 3.6 kb DNA fragment containing the *A. nidulans pyroA locus*. The fragment was then transformed into the selected deletion transformant, TSSP1.1, to generate a prototroph, TSSP25.1.

### 2.4. Generation of the *Aspergillus nidulans urdA* Complementation Strain 

A complementation strain, TSSP27.1, was obtained by transforming the ∆*urdA* strain with the *A. nidulans urdA* wild-type allele. The complementation vector was designed as follows: A DNA fragment containing the entire *urdA* coding region plus 1.5 kb of 5′ and 1.6 kb of the 3′ UTRs was PCR amplified with primers ANurdA-comF-NotI and ANurdA-comR-SpeI using genomic DNA of a FGSC4 strain as template. The PCR product was digested with *Not*I and *Spe*I and ligated into pSM3, previously digested with the same restriction enzymes. The pSM3 vector contains *pyroA* as transformation marker. The resulting plasmid was denominated as pSSP8.1. Protoplasts of strain TSSP1.1 were then transformed with an aliquot (1 µg) of this vector**. Transformants were selected on appropriate selection medium lacking pyridoxine HCl. Complementation was confirmed by a diagnostic PCR.

### 2.5. Heterologous Complementation

*Aspergillus nidulans urdA* shows 48% of identity at protein level with its putative ortholog in *A. flavus*. The entire coding region of *A. flavus urdA* (AFLA_113110), plus 1 kb 5′ UTR and 0.9 kb 3′ UTR was PCR amplified using primers AflurdA-comF-NotI and AflurdA-comR-SpeI and genomic DNA of an *A. flavus* CA14 strain as a template. The DNA fragment was digested with *Not*I and *Spe*I and cloned into pSM3 previously digested with same enzymes. Protoplasts of strain TSSP1.1 were transformed with the resulting complementation vector, pSSP9.1. Complementation was confirmed by PCR using the same primers. The strain was named TSSP28.1.

### 2.6. Morphological Studies

To evaluate the effect of *urdA* on *A. nidulans* development, the wild-type, ∆*urdA* and *urdA*-com strains were point-inoculated on solid GMM and incubated at 37 °C under continuous light and dark conditions for 7 days. Experiments were performed in triplicate. Micrographs were obtained using a Leica MZ75 dissecting microscope attached to a Leica DC50LP camera (Leica Microsystems, Buffalo Grove, IL, USA). Ethanol (70%) was sprayed on plates to improve visualization of cleistothecia prior to capturing micrographs. An additional experiment was performed to assess conidial and cleistothecial production. Approximately 5 × 10^6^ spores of wild-type, ∆*urdA* and *urdA*-com were top-agar inoculated onto 25 mL of solid GMM. After 36 h and 48 h of incubation, cores were collected for quantification of conidia (7 mm core diameter) and cleistothecia (16 mm core diameter). Cores for conidial counts were homogenized in water and spores were counted using a Hemocytometer (Hausser Scientific, Horsham, PA, USA) under a Nikon Eclipse E-400 microscope (Nikon, Melville, NY, USA). 

### 2.7. Overexpression of *brlA* in a *∆urdA* Background

The *brlA* overexpression plasmid pSSP6.1 [42] containing an inducible *alcA* promoter, *brlA* coding region and *gpdA* terminator was transformed into TSSP1.1 and TRV50 strains; the transformants were designated as TSSP26.1 and TSSP23.1 respectively. Transformants were screened by diagnostic PCR using primers AN_alcA(P)_F & ANbrlA_R., to confirm the presence of the *brlA* overexpression cassette.

For overexpression analysis using the *alcA* promoter, approximately 10^6^/mL spores of wild-type, ∆*urdA*, OE*brlA* and OE*brlA*-∆*urdA* were inoculated in 500 mL liquid GMM. The cultures were incubated for 16 h at 250 rpm and 37 °C. Mycelia were then collected and equal amounts (2 g) of biomass were rinsed with liquid TMM and shifted onto solid TMM to induce *alcA*(p). The TMM cultures were further incubated for 12 days after the shift.

### 2.8. Toxin Analysis

The wild-type and ∆*urdA* strains were top-agar inoculated onto 25 mL of solid GMM with 5 × 10^6^ spores/mL. The cultures were incubated for 36 h and 48 h. Three 16-mm diameter cores per plate were collected and extracted with chloroform for each replicate. The overnight dried extracts were resuspended in 200 µL chloroform. Twenty microliters of each sample were separated using thin-layer chromatography (TLC) as previously described [43] and analyzed for ST on silica gel plates using benzene and glacial acetic acid [95:5(*v*/*v*)] as solvent system. Aluminum chloride (15% in ethanol) was then sprayed and the plates were baked for 10 min at 80 °C. ST bands were visualized under UV light (375 nm). The ST standard was purchased from Sigma-Aldrich (St. Louis, MO, USA). 

### 2.9. Penicillin Analysis

*Bacillus calidolactis* strain C953 was used as a test organism to perform a penicillin bioassay as previously described [44] with some modifications. Fungal spores (10^6^/mL) were inoculated in 25 mL seed culture medium and incubated at 26 °C for 24 h at 250 rpm. Mycelia were then transferred to PN-inducing medium [44]. Three replicates were used in this experiment. The cultures were filtered using Miracloth (Calbiochem, San Diego, CA, USA) and supernatants were collected for analysis.

Three hundred milliliters of Tryptone-Soy Agar was inoculated with 20 mL of *B. calidolactis* C953 culture and plated on 150-mm diameter Petri plates. Twenty microliters of culture supernatant were added to 7-mm diameter wells perforated on the Trypton-soy agar medium. Bacterial cultures were incubated at 55 °C for 16 h, and inhibition halos were measured. To evaluate whether the antibacterial activity was due to the presence of penicillin or to some other fungal compound present in the supernatant, commercial penicillinase from *Bacillus cereus* (Sigma, St. Louis, MO, USA) controls were also included in this experiment. 

### 2.10. Fluorescence Microscopy

A ∆*urdA* strain bearing the construct *veA::gfp::pyrG^A. fumigatus^* was generated as follows: primers AnidveA_P7 and ANVeASTagP4 (Table S2) were used to PCR amplify a 6.6 kb DNA fragment, containing a *veA::gfp::pyrG^A. fumigatus^* fragment from *A. nidulans* T-17 strain [30]. The PCR product was then transformed into *A. nidulans* TSSP7.1 and TSSP4.1 strains (Table S1). Selected transformants were confirmed by PCR using primers VeAFnest and Gfp-mid-R. The resultant transformants were designated as TSSP3.1 and TSSP6.1 respectively. Conidia from these two strains were allowed to germinate on coverslip surfaces immersed in Watch minimal medium [45] in the dark for 6 h and then shifted to light. Samples were washed with 1× PBS after 24 h and stained with DAPI (60 ng/mL) in 0.1% Triton X-100 and 50% glycerol. A Leica DMI-6000b inverted microscope containing Nomarski optics and fluorochromes from Semrock were used to observe for green fluorescent protein (GFP) (excitation, 470; emission, 525). Hamamatsu ORCA-ER (Hamamatsu, Skokie, IL, USA) was used to take micrograph images. The exposure time for DAPI and GFP was 40 ms and 700 ms respectively.

### 2.11. Construction of a Double ∆*urdA*∆*veA* Strain

To study the epistatic relation between *veA* and *urdA*, a double deletion mutant strain ∆*urdA∆veA* (TSSP13.1) was generated by transformation of *A. nidulans* ∆*veA::pyroA* (TXF3.1) protoplasts with a *urdA* deletion cassette containing the *pyrG* marker (generated as described above). The wild-type, ∆*urdA*, ∆*veA* and ∆*urdA*∆*veA* strains were point inoculated on GMM and incubated at 37 °C under continuous light and dark for 6 days. Experiments were performed in triplicate. Micrographs were obtained using Leica MZ75 dissecting microscope attached to a Leica DC50LP camera. Quantification of conidia and cleistothecia was carried out as described above. Sixteen millimeter cores were also collected to analyze ST production as described above. 

### 2.12. Gene Expression Analysis

Mycelia were collected from top-agar inoculated cultures. Total RNA was extracted after lyophilizing the mycelia using RNeasy Mini Kit (Qiagen, Germantown, MD, USA), following manufacturer’s instructions. Quantitative Reverse Transcription-PCR (RT-qPCR) analysis was used for gene expression analysis. RQ1 DNAse (Promega, Madison, WI, USA) was used to treat 5 µg of total RNA to remove possible DNA contamination. Approximately 1 µg of DNAse treated RNA was used for cDNA synthesis using Moloney murine leukemia virus (MMLV) reverse transcriptase (Promega). Quantitative Reverse Transcription-PCR (RT-qPCR) was performed with SYBR green Jumpstart *Taq* Ready Mix (Sigma) using Mx3000p thermocycler (Agilent Technologies, Santa Clara, CA, USA). Relative expression levels were normalized to the *A. nidulans* 18S rRNA Ct values following the 2^−ΔΔCt^ method [46].

### 2.13. Statistical Analysis

All the quantitative data in this study were analyzed using ANOVA (analysis of variance) in combination with Tukey’s post hoc test using the R version 64 3.3.0 statistical software program. The significant difference among the quantitative data was recorded if the *p*-value was determined to be less than 0.05 (*p* < 0.05).

## 3. Results

### 3.1. *UrdA* Orthologs Are Found Exclusively in the Order Eurotiales

The information retrieved from aspgd.org (confirmed in fungidb.org) predicts that *urdA* (ANID_04394), located in chromosome III, encodes a 371 amino acid protein product that contains a putative helix-loop-helix (HLH) DNA binding domain (146D-246Q) which corresponds to the 2570–2737 nucleotide region. A BLAST analysis was carried out using the amino acid sequence of *A. nidulans* UrdA as the query. Putative orthologs of UrdA were found exclusively in species belonging to the order Eurotiales (within the class Eurotiomycetes), indicating a late emergence of this putative transcriptional regulator in evolution. Coverage and expected values of these putative orthologs are shown in Figure S1. The phylogenetic tree shows that orthologs from species of the genera *Penicillium*, *Rasamsonia*, *Byssochlamys* and *Talaromyces* clustered in one clade, while orthologs from *Aspergillus* species clustered in four additional clades, with those from *A. calidostus*, *A. nidulans*, *A. ochraceoroseus*, *A. rambelli*, *A. sydowii* and *A. versicolor* being the most divergent ones (Figure S2).

**Figure 1:**
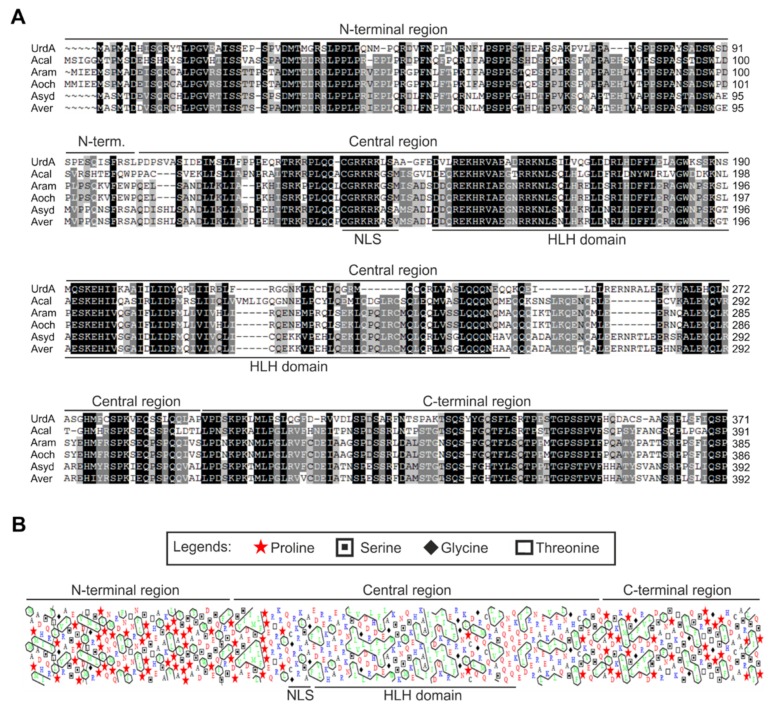
Multiple Sequence Alignment of UrdA orthologs from *Aspergillus* species. Sequences from orthologs of (**A**) *A. nidulans* (UrdA), *A. Calidoustus* (Acal), *A. rambellii* (Aram), *A. ochraceoroseus* (Aoch), *A. sydowii* (Asyd) and *A. versicolor* (Aver) were aligned using Clustal Omega and visualized using Genedoc (see Materials and Methods). The extension of the predicted N-terminal, central and C-terminal regions, as well as the putative nuclear localization signal (NLS) and helix-loop-helix (HLH) domains are indicated by black lines. (**B**) Hydrophobic cluster analysis (HCA) of UrdA. Residues in green indicate hydrophobic clusters and, thus, possible secondary structures. Residues in blue are positively charged amino acids while those in red are negatively charged. Proline, serine, glycine and threonine are designated by symbols. See Materials and Methods for additional details.

**Figure 2:**
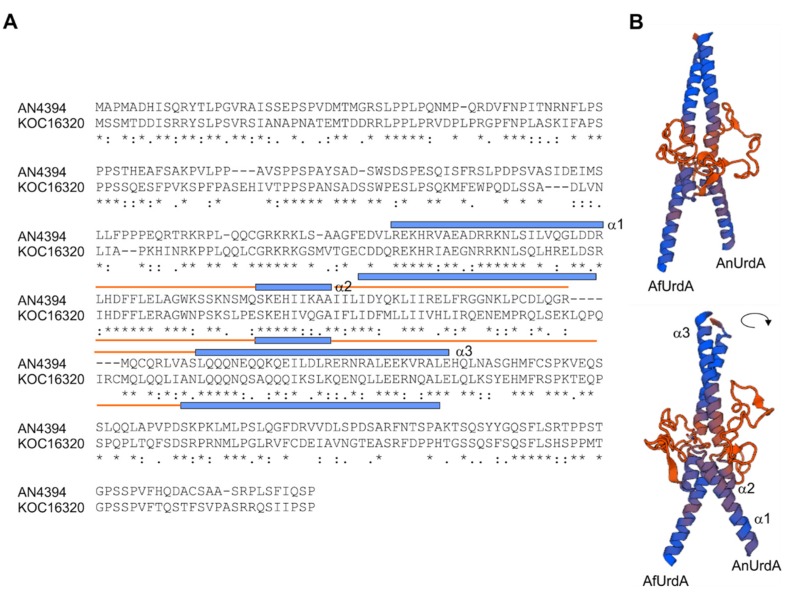
3D modeling of UrdA sequence. (**A**) Sequence alignment of AN4394/AnUrdA and its *A. flavus* ortholog KOC16320/AfUrdA. The position and extension of the three predicted α-helices of the putative HLH transcriptional regulatory domain are indicated by blue lines while orange lines delimit the extension of the loops. (**B**) Swiss-model images of a hypothetic dimer formed by the HLH domains of AnUrdA and AfUrdA. Helices are shown in blue and loops in orange.

The orthologs of the latter six species were aligned and further analyzed with the aim of identifying conserved domains (Figure 1). Overall, the polypeptide could be divided into three main regions (Figure 1A,B). N- and C-terminal domains (residues 1–91 and 294–371, respectively) were disordered regions (not shown) that contained multiple conserved proline and serine residues (see the hydrophobic cluster analysis, HCA, http://mobyle.rpbs.univ-paris-diderot.fr/cgi-bin/portal.py?form=HCA#forms::HCA, in Figure 1B), suggesting that they are prone to interact with other proteins. The central region (amino acids 103–293) is an ordered, globular domain with a predicted coiled-coil region between residues 229 and 276 (Eukaryotic Linear Motif prediction; www.elm.eu.org; not shown). This region contains multiple conserved leucine and isoleucine residues which, according to the HCA analysis, are clustered (Figure 2), purportedly furthering the formation of secondary structures such as α-helices (see below). The central region is predicted to include the HLH-type transcriptional regulatory domain (residues 146-244) and, according to NLStradamus and NLSmapper algorithms (http://www.moseslab.csb.utoronto.ca/NLStradamus/ and http://nls-mapper.iab.keio.ac.jp), a monopartite nuclear localization signal (NLS) between residues 133 and 141 (see the positively charged residues of the hypothetic NLS in Figure 1A,B).

Using Swiss-Model server (https://swissmodel.expasy.org/), we modeled a hypothetical 3D structure of a dimer composed of *A. flavus* (Af) and *A. nidulans* (An) UrdA homologs (Figure 2). Through multiple target procedures, three models were constructed based on structures of Myc-MAD and MAD-MAX heterodimers. Only the DNA-binding domain of HLH type was possible to model and in all cases predicted three α-helices (α1, α2 and α3, blue color in Figure 2B) linked by lower conserved loops (orange lines in Figure 2B). Residues located in α1 would interact with bases of the DNA. The REKHRVAEADRRKNLS sequence in AnUrdA, conserved in AfUrdA, may as well include those residues, probably arginines and lysines, whose side chains might be maintaining contacts with DNA. α3 predictably participates in the formation of the dimer. Conservation outside the HLH domain is lower among *Aspergillus* proteins (see below) and almost inexistent when compared to Myc and MAD/MAX proteins. 

### 3.2. *urdA* Is Required for Normal Conidiation in *A. nidulans*

**Figure 3:**
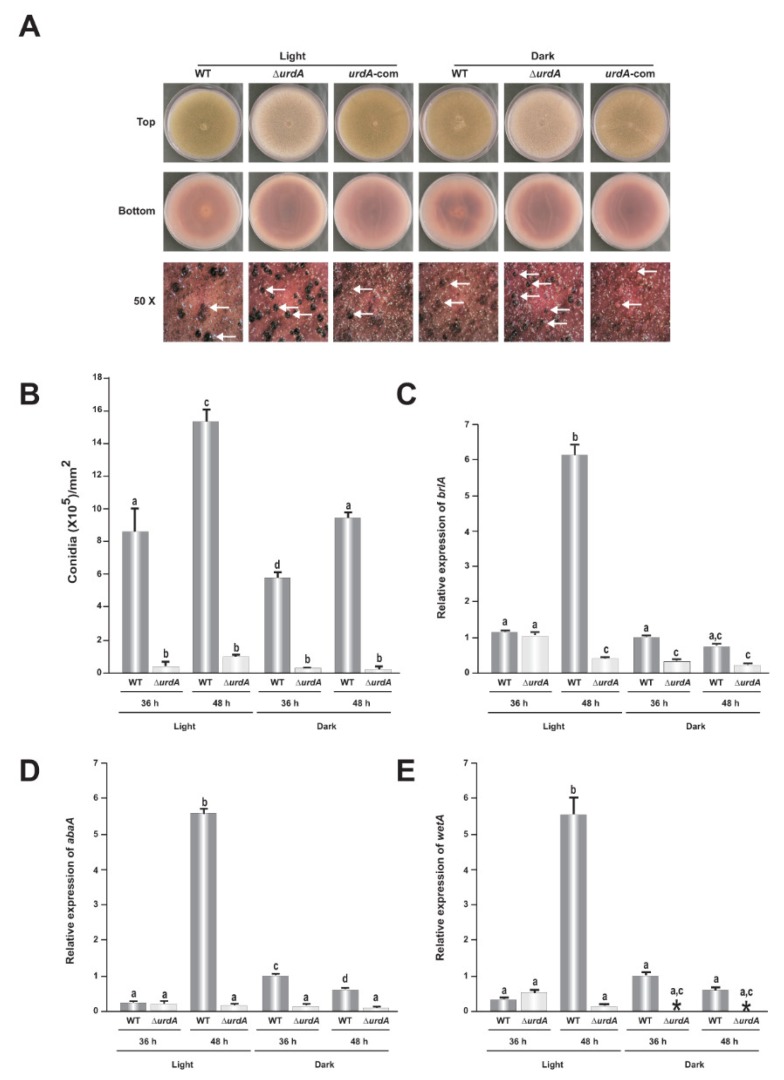
*urdA* promotes asexual development. (**A**) Wild type (WT), Δ*urdA* and *urdA*-complementation (*urdA*-com) strains were point-inoculated on glucose minimal medium (GMM) plates and incubated for 7 days in light and dark. Micrographs were taken with a Leica MZ75 dissecting microscope attached to a Leica DC50LP camera at 50× magnification after spraying the plates with 70% ethanol to enhance visualization of cleistothecia. The white arrows indicates the cleistothecia. (**B**) Quantification of conidiophores in 36 h and 48 h cultures grown on solid GMM in light and dark. Top-agar inoculated cultures were used to analyze the expression of *brlA* (**C**) *abaA* (**D**) and *wetA* (**E**). Values are means of three replicates and Error bars indicate standard errors. Different letters above the bar graphs represent significantly different values (*p* ≤ 0.05).

To study the role of UrdA in morphogenesis and other cellular processes in the *veA*+ wild-type background, a deletion strain (Δ*urdA*) and a complementation (com) strain were constructed. The deletion strain was confirmed by diagnostic PCR (Figure S3). Re-introduction of the *A. nidulans urdA* wild-type allele in the Δ*urdA* strain generated the complementation strain, which was verified by PCR (Figure S4) as described in the Materials and Methods section. The complementation strain presented wild-type phenotype (Figure 3A). In our study, Δ*urdA* produced notably fewer conidia compared to the wild-type; conidial production at 36 h was ~19-fold and 57-fold reduced in the ∆*urdA* cultures with respect to those of the wild-type strain growing in continuous light and dark, respectively. These differences continued over time, with a ~14-fold decrease in conidial production in the light and 31-fold in the dark after 48 h (Figure 3B). In addition, a reduction of *brlA*, *abaA* and *wetA* expression was observed in the ∆*urdA* mutant with respect to the control, particularly in 48 h-old light cultures (Figure 3C–E). 

### 3.3. Overexpression of *brlA* Is Not Sufficient to Induce Conidiation in the Absence of *urdA*

**Figure 4:**
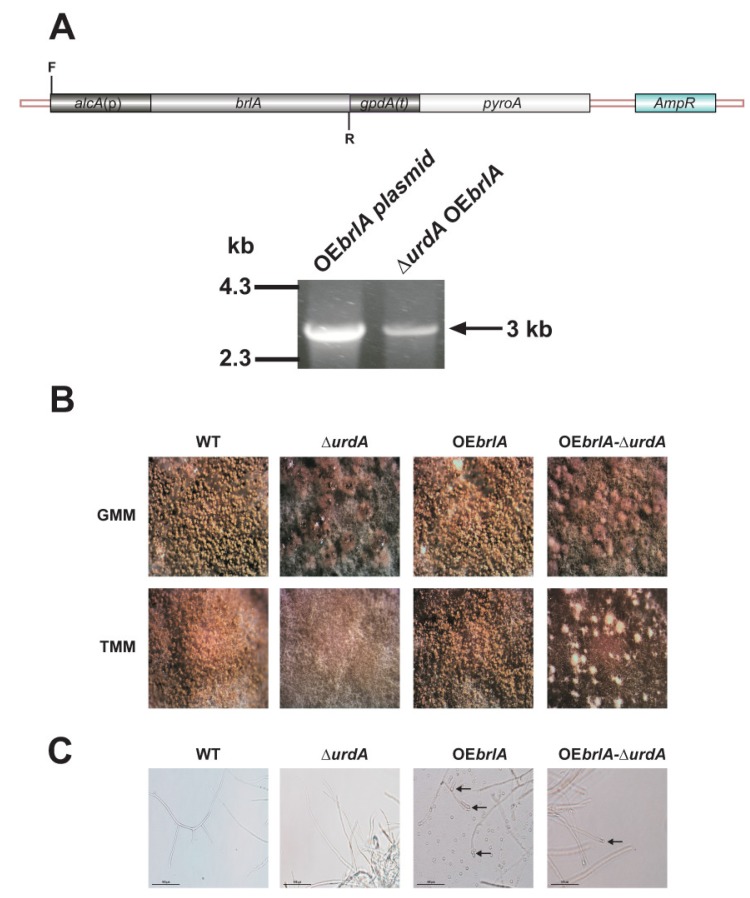
Overexpression of *brlA* is not sufficient to induce conidiation in the absence of *urdA*. (**A**) *Aspergillus nidulans* wild type (TRV50) and Δ*urdA* strains were transformed with an overexpression plasmid containing *A. nidulans brlA* coding region driven by the inducible promoter *alcA*. Diagnostic PCR was used to confirm the incorporation of the overexpression plasmid into the genome of the Δ*urdA* host strain after transformation, using primers AN_alcA(P)_F & ANbrlA_R (Table S2), labeled in this figure as F and R respectively. The expected 3 kb PCR product was obtained. (**B**) *A. nidulans* wild type (WT), Δ*urdA*, OE*brlA* and Δ*urdA*-OE*brlA* strains were inoculated with 10^6^ conidia/mL in liquid GMM at 250 rpm for 18 h and then shifted onto solid GMM or TMM. Micrographs were taken after 12 days with a Leica MZ75 dissecting microscope attached to a Leica DC50LP camera at 50× magnification. (**C**) One gram of mycelium was also inoculated in 50 mL of liquid TMM and grown at 37 °C for 18 h after shift to observed possible formation of conidia in submerged cultures. Black arrows show formation of conidia from the tip of hyphae.

Based on the finding that *urdA* affected conidiation, we analyzed whether overexpression of *brlA* was sufficient to rescue asexual development in the absence of *urdA* in a strain with an intact *veA locus*. Diagnostic PCR confirmed the presence of *alcA*(p)::*brlA* construct in the transformed ∆*urdA* strain (Figure 4A). Our results indicated that the overexpressed *brlA* in an *urdA* deletion background did not rescue wild-type conidiation (Figure 4B), since only few conidia were observed (Figure 3C). Similar results were obtained in submerged cultures, where the *alcA*(p)::*brlA* *∆urdA* strain produced few conidia at the tip of some hyphae (Figure 4C). 

### 3.4. Effect of *urdA* on Sexual Development

**Figure 5:**
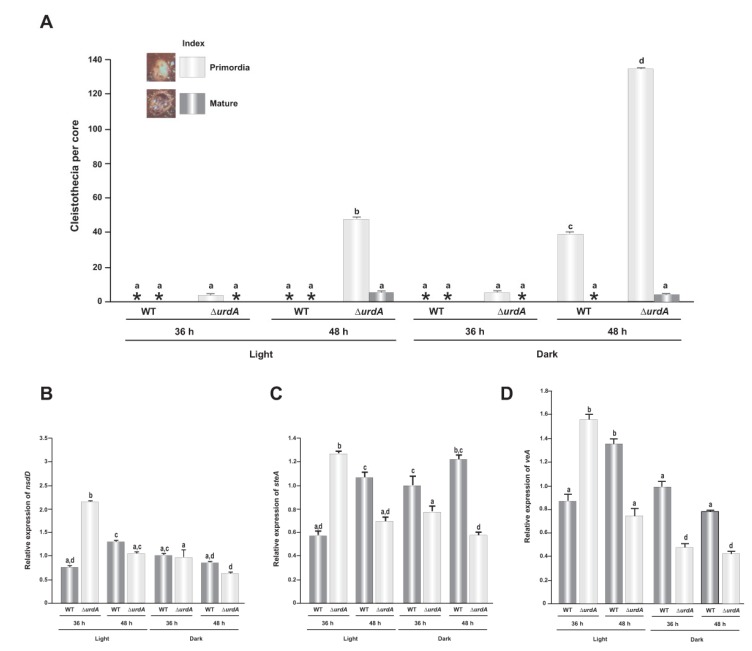
*urdA* represses sexual development. (**A**) Quantification of cleistothecia (both immature and mature) in 36 h and 48 h cultures grown on solid GMM in light and dark. Asterisks indicate not detected. Top-agar inoculated cultures were used to analyze the expression of *nsdD* (**B**) *steA* (**C**) and *veA* (**D**). Values are means of three replicates and error bar indicates standard errors. Different letters above the bar graphs represent significantly different values (*p* ≤ 0.05).

The *A. nidulans* ∆*urdA* strain presented a precocious and increased sexual development as compared to the wild-type control (Figure 5A). Cleistothecial primordia were already present in ∆*urdA* cultures after 36 h of incubation, in both light and dark cultures, while they were absent in the wild type. At 48 h, cleistothecia were present in ∆*urdA* cultures grown in the light and in the dark, some of them already showing pigmentation, while in the wild-type only cleistothecial primordia were observed at that time point, and only in dark cultures. Our gene expression analysis revealed that expression of *nsdD*, encoding a transcription factor necessary for sexual development [47] increased in the ∆*urdA* strain at 36 h and under light conditions (Figure 5B). In addition, expression of *steA*, a gene encoding another transcription factor necessary for cleistothecial formation and ascosporogenesis [48], as well as expression of *veA*, was more than 2-fold higher in the *urdA* deletion mutant with respect to the control at 36 h, before the levels decreased over time (Figure 5C). After 7 days ∆*urdA* cultures showed abundant fully pigmented cleistothecia in both light and dark, whereas the wild type formed few cleistothecia under those conditions (Figure 5A).

### 3.5. Heterologous Complementation of *A. nidulans* ∆*urdA* with the Putative *urdA* Ortholog of *A. flavus* Does Not Fully Rescue Wild-Type Morphological Phenotype

**Figure 6:**
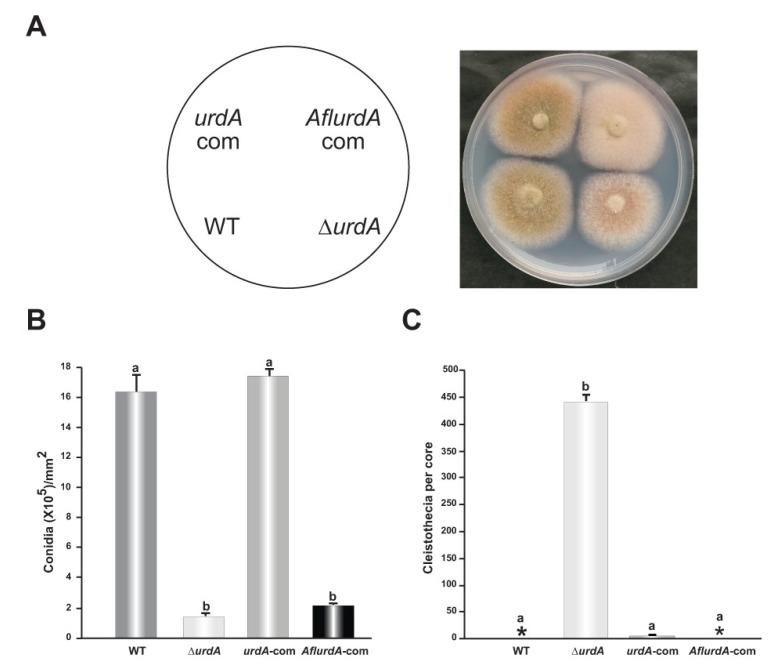
Heterologous complementation of *A. flavus urdA* in the *A. nidulans* Δ*urdA* strain. (**A**) *A. nidulans* and *A. flavus urdA* complementation strains (*urdA*-com and *AflurdA*-com respectively), together with the Δ*urdA* and wild type (WT) control were point-inoculated on GMM and incubated for 4 days at 37 °C in the light. (**B**) Quantification of conidia. Cores (7 mm diameter) were collected 1 cm from the colony center, homogenized in water and counted under the microscope. (**C**) Quantification of cleistothecia. Cores (16 mm diameter) were harvested 1 cm from the colony center and sprayed with 70% ethanol to improve visualization of fruiting bodies. Asterisks indicate not detected. Values are means of three replicates and error bars indicate standard errors. Different letters above the bar graphs represent significantly different values (*p* ≤ 0.05).

To assess possible functional conservation between *A. nidulans* UrdA and putative homologs from other *Aspergillus* species, *A. nidulans* ∆*urdA* strain was heterologously complemented with the orthologous gene from the AF-producer and agriculturally and medically important fungus *A. flavus* (*AflurdA*-com). The *A. flavus* ortholog presents 48% identity to UrdA however it was located in a different clade in the phylogenetic tree in Figure S2. The heterologous complemented strain presented a reduction in conidiation as in the ∆*urdA* strain (Figure 6A,B), indicating that the *A. flavus* ortholog gene was unable to rescue wild-type conidiation in *A. nidulans* ∆*urdA*. However, the *A. flavus* ortholog repressed cleistothecial production as in the wild type control (Figure 6C).

### 3.6. UrdA Is a Negative Regulator of Sterigmatocystin Biosynthesis and Production of Several Unknown Metabolites in a Light-Dependent Manner

**Figure 7:**
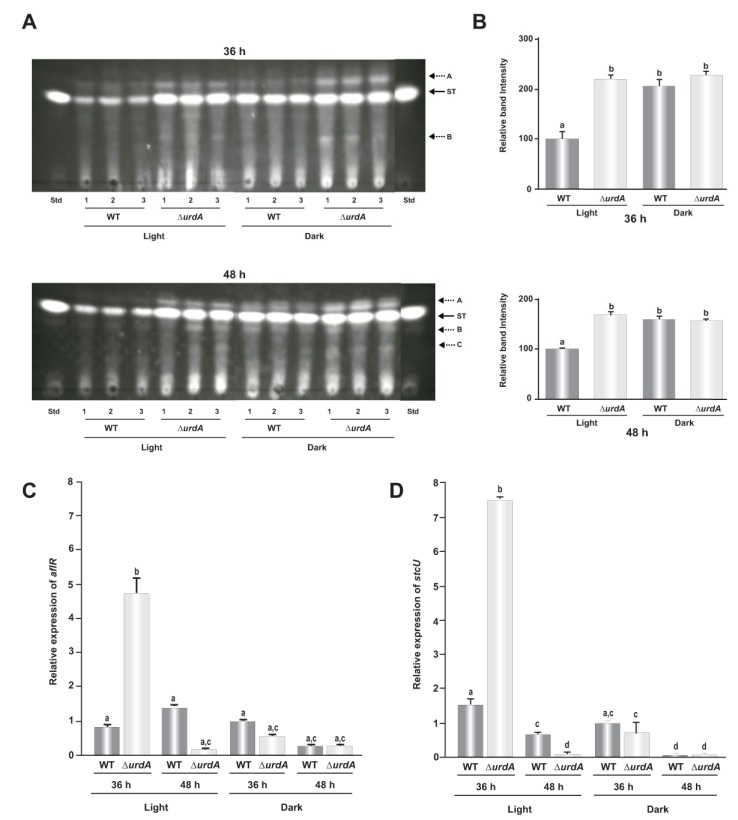
Effect of *urdA* on sterigmatocystin (ST) production in *A. nidulans*. (**A**) Thin-layer chromatography (TLC) analysis of ST produced by wild type (WT) and Δ*urdA* cultures grown on top-agar inoculated solid GMM at 37 °C in light and dark conditions for 36 h, and 48 h. ST, a commercial ST standard from SIGMA. Broken arrows indicate other metabolites whose synthesis is also affected by *urdA*. (**B**) Densitometry of the ST bands using ImageJ software [51]. The ST bands were normalized to wild-type levels in the light, considered as 100 percent. (**C**) Effect of *urdA* on *aflR* and *stcU* expression analyzed by RT-qPCR. Values are means of three replicates and error bar indicates standard errors. Different letters above the bar graphs represent significantly different values (*p* ≤ 0.05).

In this study the possible effect of UrdA on the production of ST toxin, a penultimate precursor of aflatoxin B_1_ in other *Aspergillus* spp., was also evaluated. The *A. nidulans veA+* wild-type strain produces significantly more ST in the dark than in the light when growing on solid GMM [49]. However, the Δ*urdA* strain produced as much ST, in both light and dark, as the wild-type *veA*+ dark cultures (Figure 7A,B). This suggests that the role of *urdA* in regulating ST production in *A. nidulans* is light-dependent. Interestingly, our chemical analysis also indicated that the absence of *urdA* affects the synthesis of some additional unknown metabolites (Figure 7A), suggesting a broader effect of UrdA in the control of secondary metabolism. Expression analysis of *aflR* in the *urdA* deletion mutant showed an approximate 6-fold increase with respect to that of the wild type at 36 h when the cultures were growing in the light (Figure 7C). Similarly, expression of *stcU*, a structural gene in the cluster commonly used as an indicator of cluster activation [50], was also notably increased compared to the control at 36 h in the light (Figure 7D). 

These results show that UrdA influences the synthesis of ST as well as the production of other fungal metabolites. For this reason, we also examined whether UrdA affects the biosynthesis of penicillin (PN) in *A. nidulans*. However, our experiment indicated that there is no statistically significant difference in the production of PN between wild-type and Δ*urdA* cultures (Figure S5).

### 3.7. UrdA Affects the Level of VeA in Fungal Cells

**Figure 8:**
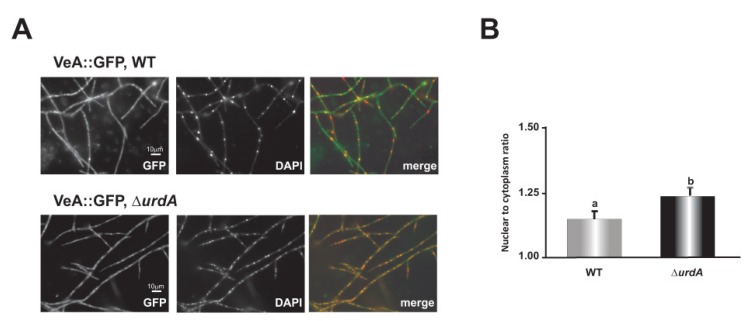
*urdA* influences accumulation and location of VeA in fungal cells. *A. nidulans* wild type (WT) and a Δ*urdA* strain containing a *veA*::gfp::*pyrG* cassette were cultured in the dark for 6 h and then shifted to light. (**A**) Micrographs showing green fluorescence, DAPI stained nuclei, and merged images. (**B**) Nuclear to cytoplasmic ratio of the fluorescence in each compartments were calculated using the pixel intensity values. The mean were taken from 40 measurements at different nuclear and cytoplasmic compartments. Different letters above the bar graphs represent significantly different values (*p* ≤ 0.05).

Figure 5D showed a significant variation in *veA* expression in the deletion *urdA* mutant. To study whether UrdA affects the abundance and subcellular localization of VeA, a *veA::gfp::pyrG^Afum^* strain was generated in wild-type and *urdA* deletion genetic backgrounds. A diagnostic PCR, with primers veAF5′UTR and veAGFP, was used to confirm the presence of the *veA::gfp::pyrG* fragment in the *veA locus*. Amplification of a 4.0 kb PCR product indicated the correct integration (Figure S6). Microscopic examination of the cultures grown in the dark for 6 h and then shifted to light revealed that the abundance of VeA was greater in the absence of *urdA* compared to the control strain (Figure 8A). This enhanced VeA accumulation was particularly notable in nuclear compartments as seen by the nucleus to cytoplasmic ratio of the fluorescence intensity (Figure 8B). A parallel set of cultures further grown in the dark did not show any differences with respect to the control (data not shown).

### 3.8. Epistatic Relationship between *urdA* and *veA*

**Figure 9:**
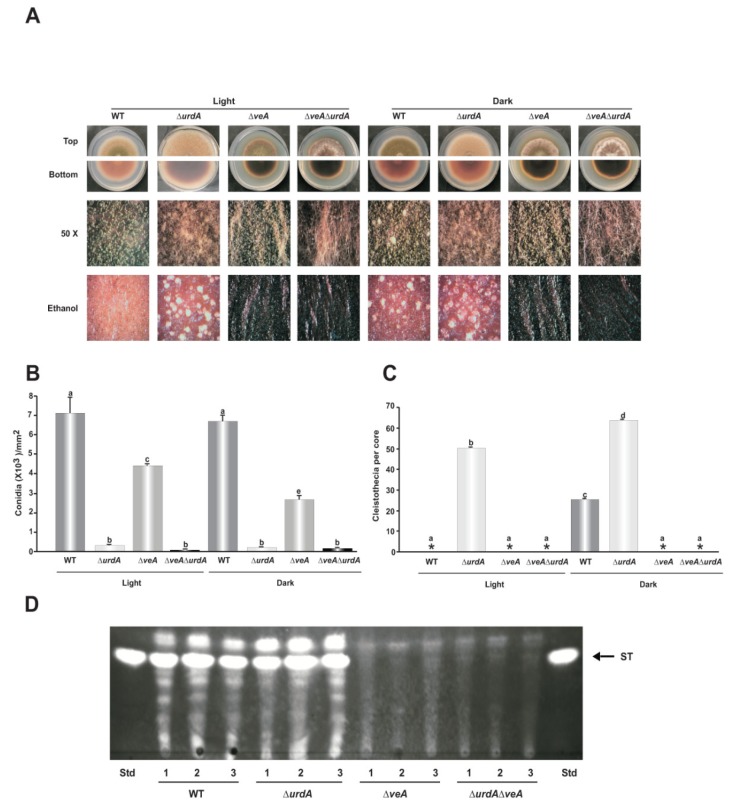
Epistatic relation between *veA* and *urdA*. (**A**) Wild type, Δ*urdA*, Δ*veA* and Δ*veA* Δ*urdA* were point-inoculated and cultivated in dark and light conditions for 6 days. Micrographs were taken with a Leica MZ75 dissecting microscope attached to a Leica DC50LP camera at 50× magnification. (**B**) Quantification of conidiospores as described in Material and Methods (**C**) Quantification of cleistothecia after spraying the plates with 70% ethanol to enhance visualization. (**D**) TLC analysis of ST. Cores (16 mm) were collected 1.5 cm from the center of the colony after 6 days and ST was extracted as described in Material and Methods. Asterisks indicate not detected. Std., Standard. Values are means of three replicates and error bars indicate standard errors. Different letters above the bar graphs represent significantly different values (*p* ≤ 0.05).

Previous studies revealed that the global regulator *veA* is required for sexual development and is a negative regulator of conidiation [19], whereas this study showed that *urdA* is a negative regulator of sexual development and a positive regulator of asexual development. We examined the epistatic relationship between *veA* and *urdA* by generating a double deletion mutant ∆*veA*∆*urdA* as described in Material and Methods. The resulting ∆*veA*∆*urdA* transformant was confirmed by a diagnostic PCR. The wild type TRV50.2 strain, single mutants ∆*urdA* and ∆*veA* along with the double mutant ∆*veA*∆*urdA* were point-inoculated on GMM plates and grown for 6 days in light and dark (Figure 9A). The double mutant ∆*veA*∆*urdA* showed a distinct phenotype, failing to produce either cleistothecia or conidia; instead it produced abundant aerial hyphae (Figure 9B,C). 

Sterigmatocystin toxin production was also analyzed in these strains. Unlike the wild type and ∆*urdA*, the double mutant did not produce ST, which coincides with the expected phenotype of the ∆*veA* mutant (Figure 9D), as it is known that *veA* is required for ST production [52].

## 4. Discussion

**Figure 10:**
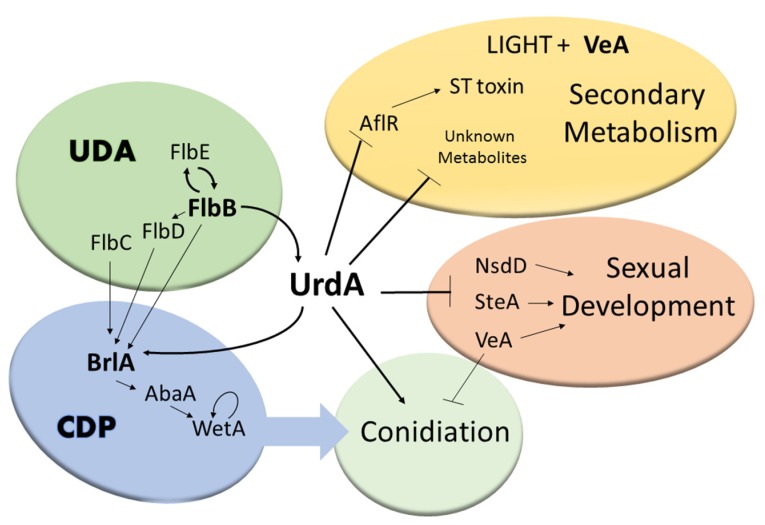
Model for UrdA activity. The upstream developmental activators (UDA) signaling pathway controls UrdA [27], and UrdA controls the central developmental pathway (CDP) pathway and conidiation (in both *veA1* [27] and in *veA* wild-type background, as shown in the present study). UrdA also negatively affects NsdD, SteA and VeA, inhibiting sexual development. In addition, UrdA represses the production of ST (by negatively influencing AflR) and of other known secondary metabolites. Protein codes are used for simplicity.

Conidiation is the most efficient form of dissemination in *Aspergillus* species. Regulators of asexual development such as BrlA, AbaA, WetA in the CDP pathway or FlbB and FlbE in the UDA pathway show variable patterns of conservation [53,54], with the presence of BrlA being restricted to the order Eurotiales. In the current study we showed that the *flbB*-dependent transcription factor gene *urdA*, controls both production of asexual spores as well as sexual development in the wild type *veA+* strain of *A. nidulans* (Figure 10). Sequence analyses showed that UrdA is conserved exclusively in species of the order Eurotiales. Thus, phylostratigrapy [55] studies strongly suggest that this putative transcription factor emerged late in evolution, developing three domains that are clearly discernible based on our alignments and HCA analyses. The HLH-type transcriptional regulatory domain (also the predicted NLS) shows the highest level of conservation among UrdA orthologs, suggesting that it may be able to bind similar consensus sequences in target promoters. However, the conservation of the disordered N- and C-terminal regions is low, resulting in a group of six orthologs (including UrdA) with the highest sequence divergence. These differences may have caused a modification of the interaction partners or the regulatory mechanisms in which UrdA is required, resulting in a rewiring of the corresponding transcriptional network(s). Absence of *urdA* resulted in a drastic reduction in conidial production while promoting abundant formation of cleistothecia, even under the light, a condition that inhibits sexual development and promotes conidiation in strains with a *veA*+ wild type allele [56]. Similarly, deletion of *ecdR*, the *urdA* homolog in *A. oryzae* with 99% identity to that of *A. flavus* at nucleotide level, produced fewer conidia and higher number of sclerotia compared to the wild-type [27,57,58]. This is relevant since, in addition to serving an important role as survival structures [59,60,61]; sclerotia are stromata for the formation of sexual structures [62,63,64]. UrdA/EcdR appear to have a conserved role in repressing cleistothecia/sclerotia production. However, heterologous complementation with the *A. flavus* putative ortholog, which presents 48% identity (at protein level) to that in *A. nidulans*, did not rescue normal conidiation in the *A. nidulans* deletion mutant, suggesting that *urdA/ecdR* from *A. flavus* and likely its ecotype *A. oryzae* [9], might present certain variations in their mechanisms of action. The sequence divergence and the above-mentioned hypothetic network rewiring events could explain, for example, why the *A. flavus* ortholog of UrdA is unable to remediate the conidiation defect of the *urdA* deletion strain of *A. nidulans* but suppresses the premature induction of sexual development.

To gain insight into UrdA mechanism of action in *A. nidulans*, we examined its role on the regulation of the CDP controlling conidiation in this fungus, specifically *brlA*, *abaA* and *wetA* [28,65]. Our results revealed that the expression of these three genes is reduced in the Δ*urdA* mutant compared to the wild type. This reduction was most drastic at 48 h in cultures exposed to light. However, a reduction of asexual spore production is already observed at 36 h, suggesting an additional early mechanism of UrdA, besides controlling the CDP, which also influences asexual development. Overexpression of *brlA* in the absence of *urdA* was unable to rescue wild-type conidiation, also suggesting that *urdA* carries out additional roles in the control of conidiation downstream to *brlA*. Coinciding with the increase and precocious sexual fruiting bodies production observed in the absence of *urdA* in the light, we detected an earlier increase in the expression of genes known to be required for sexual development, specifically *nsdD*, *steA* and *veA* [47,48]. The increase in the expression of these genes in light cultures could contribute to the phenotype observed where cleistothecia are abnormally forming early and in abundant numbers under illumination in Δ*urdA* (Figure 10). 

Morphological development and secondary metabolism are genetically linked [18,19,33,66]. *A*. *nidulans* produces small levels of ST in the light compared to those in the dark. Interestingly, ∆*urdA* produced as much ST in light culture as that of wild-type or ∆*urdA* cultures grown in the dark. These observations suggest that *urdA* is a light-dependent negative regulator of ST production (Figure 10). Furthermore, our results indicated that such an increase in ST toxin in ∆*urdA* light cultures was accompanied by high levels of *aflR* expression that resulted in cluster activation.

In addition to the effect of *urdA* on ST production, our analysis revealed other unknown metabolites accumulating at higher levels in the absence of *urdA* compared to the wild-type, indicating a broader regulatory potential. This correlates to the previous study done by Oiartzabal-Arano et al. [27] where *urdA* was shown to regulate two genes of the dba secondary metabolite gene cluster. For this reason, we also examined the role of *urdA* in PN biosynthesis and found no effect on the production of this antibiotic in *A. nidulans*. 

A well-known genetic link between morphogenesis and secondary metabolism is the global regulatory gene *veA*, [18,19,33,51,66]. This global regulator is conserved in numerous fungal species but absent in plant or animal genomes [19,51,67,68,69,70,71,72]. The subcellular localization of the VeA wild-type protein (VeA+) is light dependent [30]. While in the light VeA is abundantly present in the cytoplasm, in the dark this protein mainly accumulates in nuclei, promoting sexual development, biosynthesis of ST and other secondary metabolites, and repressing conidiation. Our study showed that absence of *urdA* increases VeA protein levels in the cell in light cultures, particularly in nuclei, possibly contributing, at least in part, to the increase in sexual development, decrease in conidiation and greater amount of ST and other compounds in light Δ*urdA* cultures. These results suggest that the synthesis of VeA and its transport to the nucleus is influenced by UrdA.

Since VeA and UrdA present antagonistic roles in *A. nidulans* with respect to sexual and asexual development, we investigated the epistatic relationship between these two regulators by the generation of a double mutant ∆*veA*∆*urdA*. This double mutant neither produced conidia nor cleistothecia, instead it grew mainly vegetatively forming abundant aerial mycelium. In addition, the medium pigmentation in the double mutant was the same as that of ∆*veA*. Although *veA* appears to be epistatic to *urdA*, the double mutant ∆*veA*∆*urdA* failed to hyperconidiate as in the case of the ∆*veA* mutant. In addition, ∆*veA*∆*urdA* did not produce ST, as in the case of ∆*veA* and unlike ∆*urdA* and the wild-type strain, which produced ST. Thus, although VeA and UrdA appear functionally related, they also perform independent regulatory roles.

In conclusion, this study provides further insight into the role and mechanism of action of UrdA, a transcription factor with a putative HLH DNA binding domain in the model filamentous fungus *A. nidulans*. The bioinformatics analysis revealed that *urdA* is specific to the order Eurotiales. UrdA function in *A. nidulans* also appears to be distinct, at least from that of *A. flavus*. In *A. nidulans*, UrdA has an important role in repressing sexual development and promoting the formation of air-borne conidia, particularly when this organism is exposed to light, a harmful environmental factor for fungal growth**. Furthermore, *urdA* influences secondary metabolism in a light-dependent manner, including the production of the carcinogenic mycotoxin ST. Importantly, UrdA affects the abundance and subcellular localization of VeA, possibly further contributing to the observed broad regulatory spectrum of UrdA in the model filamentous fungus *A. nidulans*. 

## Supplementary Materials

The following are available online at http://www.mdpi.com/2073-4425/9/12/570/s1,Figure S1: BLASTp results for UrdA. Ortholog sequences of UrdA, ordered according to the expected (e) value (color bar below). The extension of the colored lines indicates the coverage. The black arrow delimits those orthologs analyzed in the phylogenetic tree in Figure S2 and those excluded for analysis, Figure S2: Evolutionary analysis of UrdA orthologs. Phylogenetic tree for the orthologs of UrdA (Mega Software). Sequences belonging to species of the genera *Penicillium*, *Talaromyces*, *Byssochlamys* and *Rasamsonia* are located in a different clade compared to those from *Aspergilli*, which are distributed in four different clades. The purple and dotted red squares indicate closest UrdA orthologs and An4394/UrdA, respectively, Figure S3: Generation of *A. nidulans* Δ*urdA* strain. (A) Schematic representation showing deletion of *urdA* by gene replacement using *pyrG* from *Aspergillus fumigatus*. The crosses (X) indicate recombination events between the homologous flanking regions. (B) Diagnostic PCR confirming deletion of *urdA* in the selected transformants using primers urdA-P0 and pyrG_Afum_R denoted by F and R respectively (Table S2). Deletion of Δ*urdA* is indicated by the presence of the 3.3 kb DNA fragment, Figure S4: Generation of *A. nidulans* and *A. flavus* complementation strains. The *A. nidulans* Δ*urdA* strain was transformed with plasmids containing *urdA* from *Aspergillus nidulans* and its *Aspergillus flavus* homolog respectively. (A) Linear diagram corresponding to the *A. nidulans urdA* complementation vector. (B) Diagnostic PCR was used to confirm the reintegration of *urdA* in the genome using primers ANurdA-comF-NotI and ANurdA-comR-SpeI (Table S2), labeled in this figure as F and R respectively. The expected 4.4 kb PCR product was obtained. (C) Confirmation of the integration of the *A.flavus urdA* into the genome of the *A. nidulans* Δ*urdA* host strain was carried out using a similar strategy with primers AflurdA-comF-NotI and AflurdA-comR-SpeI (Table S2). The expected 3.7 kb PCR product was obtained, Figure S5: *urdA* does not affect penicillin (PN) production in *A. nidulans*. Extracts of wild type (WT) and Δ*urdA* were analyzed for penicillin presence using the bioassay described in Materials and Methods. Diameter of PN growth inhibition halos in mm is shown. Values shown are means of triplicates. Error bars represent standard error, Figure S6: Generation of the *veA::gfp* and *veA::gfp* Δ*urdA* strains. (A) The *veA::gfp::pyrG^A.fumigatus^* and *veA::gfp::pyrG^A.fumigatus^* Δ*urdA* strains was generated as follows: primers AnidveA_P7 and ANVeASTagP4 (Table S2) were used to PCR amplify a 6.6 kb fragment containing a *veA::gfp::pyrG^A. fumigatus^* fragment from *A. nidulans* T-17 strain (Stinnett et al., 2007). The PCR product was then transformed into *A. nidulans* TSSP7.1 and TSSP4.1 strains (Table S1). (B) Diagnostic PCR was used to confirm the integration of *veA*::*gfp* in the host strains after transformation, using primers VeAFnest and Gfp-mid-R. The expected 4.0 kb PCR product was obtained. Table S1: Fungal strains used in this study, Table S2: Primers used in this study.

## Appendix

**Table A1:** Supplementary Strains Table.

Strain	Pertinent Genotype	Source
FGSC4	Wild type	FGSC*
BD834	*∆urdA::pyrG^A. fum^; pyrG89; pyroA4*	This study
TRV50.2	Wild type	[73]
TRV50	*pyroA4*, *∆nkuA*::*argB*; *veA*+	[73]
T-17	*veA::gfp:: pyrG^A.fum^; pyrG89; pyroA4*	[30]
RJMP1.49	*pyrG89; argB2; ∆nku::argB; pyroA4*	[74]
TXF3.1	*pyrG89; argB2; ∆nku::argB; ∆veA::pyro; pyroA4*	[75]
TXFp2.1	*∆veA::pyrG^A.fum^; pyrG89; argB2; ∆nku::argB; pyroA4,*	[75]
TSSP1.1	*∆urdA::pyrG^A.fum^; pyrG89; argB2; ∆nku::argB; pyroA4;*	This study
TSSP3.1	*veA::* *gfp::pyrG^A.fum^; pyrG89, ∆nku::argB; argB2;*	This study
TSSP4.1	*pyrG89, argB2; ∆nku::argB; ∆urdA::pyroA pyroA4*	This study
TSSP6.1	*pyrG89; veA::gfp::pyrG^A.fum^; ∆nku::argB; argB2; ∆urdA::pyroA; pyroA4;*	This study
TSSP7.1	*pyrG89; argB2; ∆nkuA::argB*	[75]
TSSP13.1	*pyrG89; ∆urdA::pyrG^A.fum^ argB2; ∆nku::argB; ∆veA::pyroA; pyroA4*	This study
TSSP23.1	*pyrG89; argB2; ∆nkuA::argB; alcA::brlA::gpdA::pyroA; pyroA4,*	This study
TSSP25.1	*∆urdA::pyrG ^A. fum^;pyrG89;argB2; ∆nkuA::argB*	This study
TSSP26.1	*∆urdA::pyrG ^A. fum^; pyrG89; ∆nkuA::argB; argB2;; alcA::brlA::gpdA::pyroA; pyroA4*	This study
CA14	*niaD-;pyrG-; Δku70*	USDA**
TSSP27.1	*∆urdA::pyrG^A.fum^; pyrG89; ∆nkuA::argB: argB2; urdA*::*pyro; pyroA4*	This Study
TSSP28.1	*∆urdA::pyrG ^A. fum^; pyrG89; ∆nkuA::argB; argB2; urdA^A.flavus^*::*pyroA;pyroA4*	This Study

*FGSC, Fungal Genetics Stock Center. **USDA, United State Department of Agriculture
