# Supplementary material for: UrdA Controls Secondary Metabolite Production and the Balance between Asexual and Sexual Development in Aspergillus nidulans
Source: Genes (Basel). 2018 Nov 23;9(12):570. doi: 10.3390/genes9120570 (PMC6316066; doi:10.3390/genes9120570)
Supplement: Supplementary file 1 [file genes-09-00570-s001.zip › genes-382948-supplementary-figures.docx]

Supplementary figures


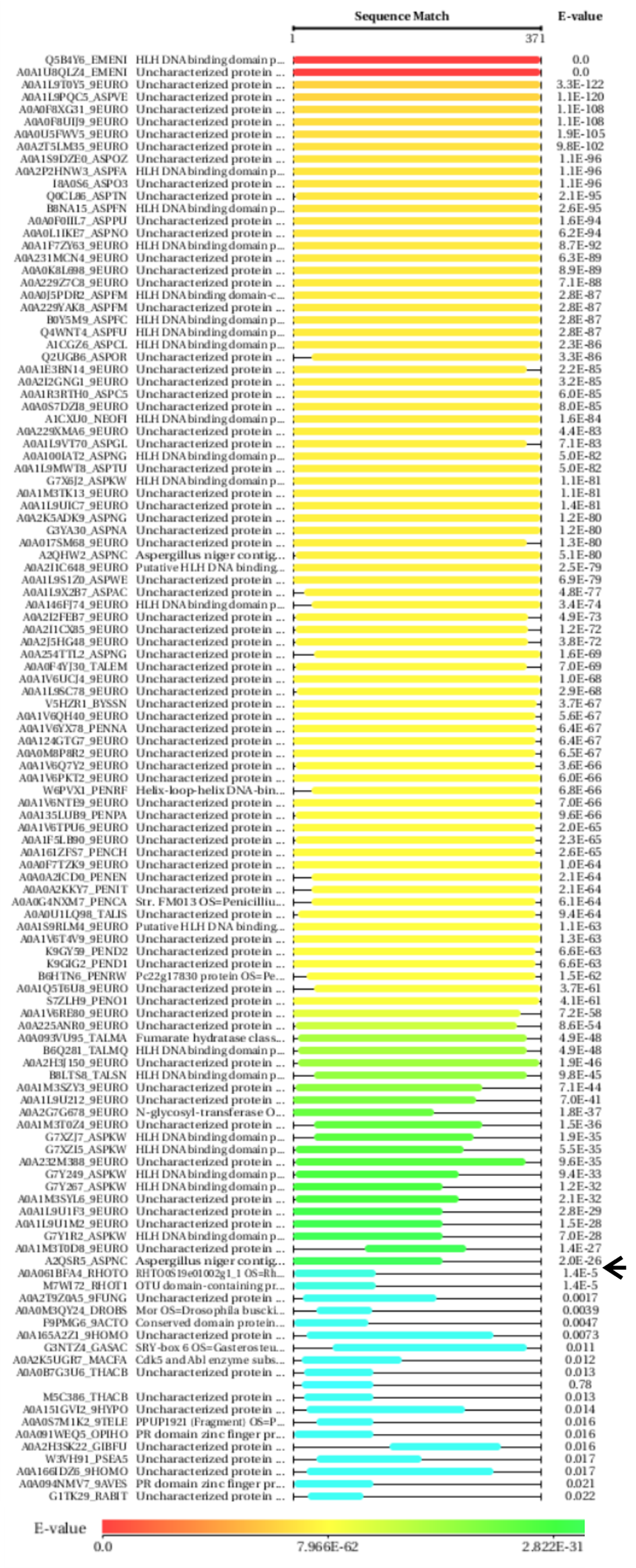


**Figure S1. BLASTp results for UrdA.** Ortholog sequences of UrdA, ordered according to the expected (e) value (color bar below). The extension of the colored lines indicates the coverage. The black arrow delimits those orthologs analyzed in the phylogenetic tree in Figure S2 and those excluded for analysis.


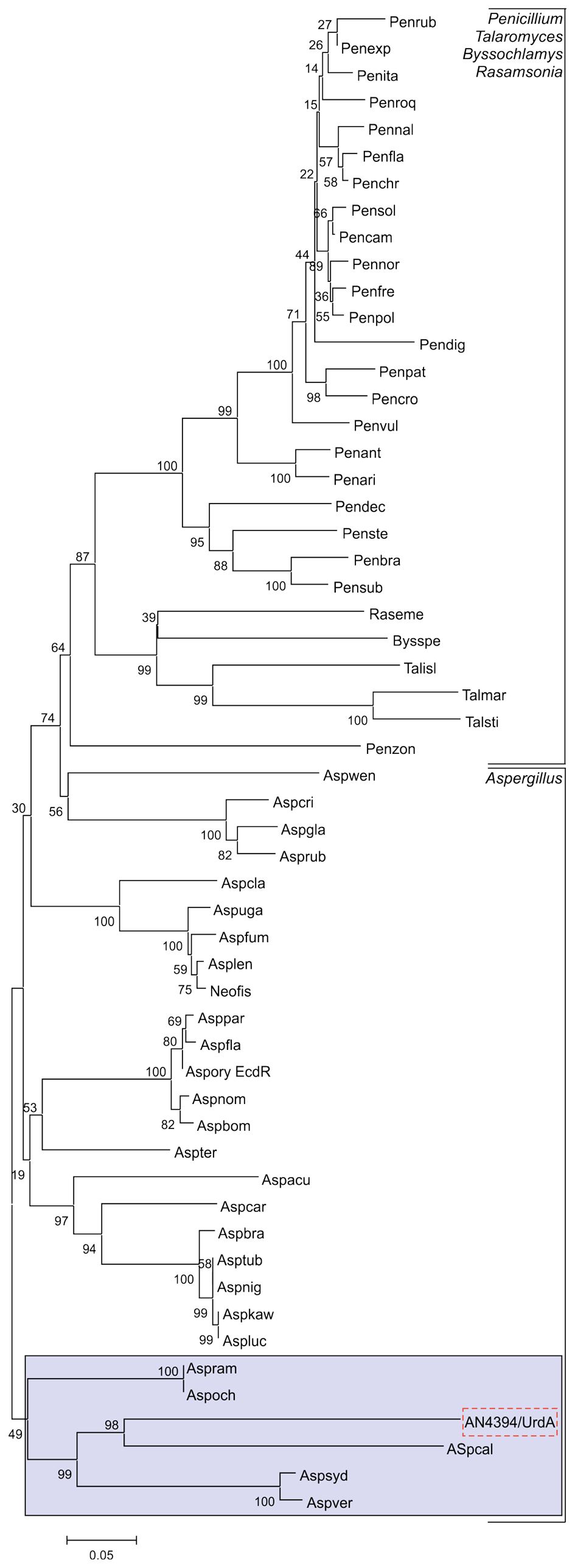


**Figure S2. Evolutionary analysis of UrdA orthologs.** Phylogenetic tree for the orthologs of UrdA (Mega Software). Sequences belonging to species of the genera *Penicillium*, *Talaromyces*, *Byssochlamys* and *Rasamsonia* are located in a different clade compared to those from *Aspergilli*, which are distributed in four different clades. The purple and dotted red squares indicate closest UrdA orthologs and An4394/UrdA, respectively.


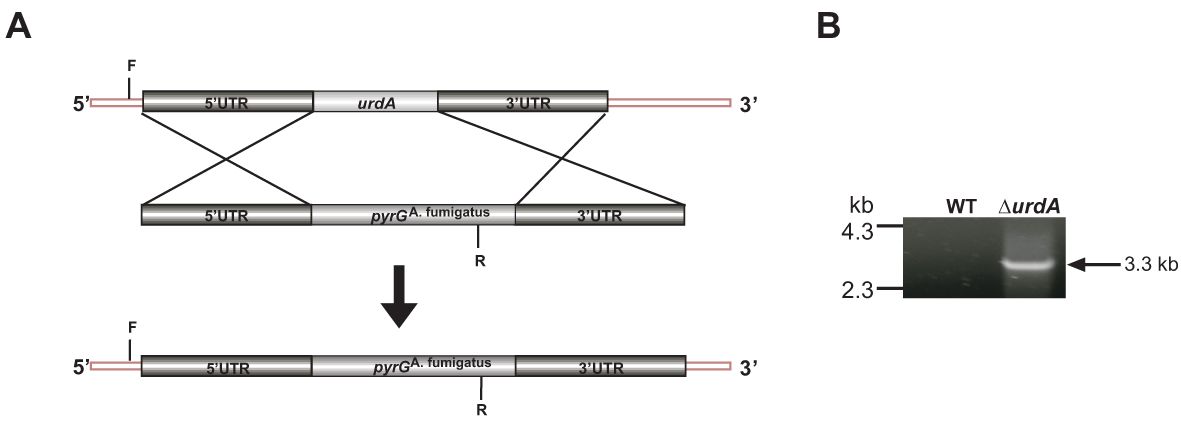


**Figure S3. Generation of *A. nidulans* Δ*urdA* strain.** (A) Schematic representation showing deletion of *urdA* by gene replacement using *pyrG* from *Aspergillus fumigatus*. The crosses (X) indicate recombination events between the homologous flanking regions. (B) Diagnostic PCR confirming deletion of *urdA* in the selected transformants using primers urdA-P0 and pyrG_Afum_R denoted by F and R respectively (Table S2). Deletion of Δ*urdA* is indicated by the presence of the 3.3 kb DNA fragment.


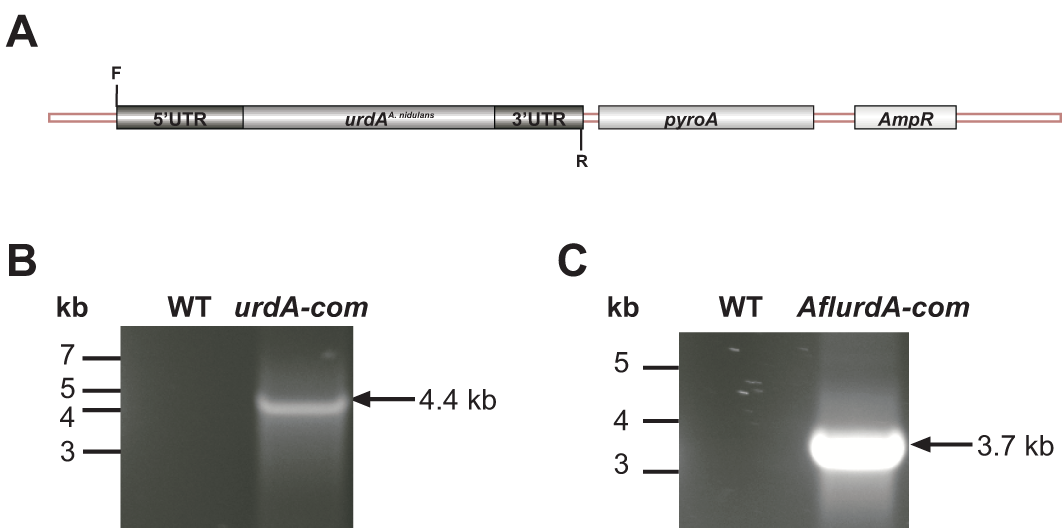


**Figure S4. Generation of *A. nidulans* and *A. flavus* complementation strains.** The *A. nidulans* Δ*urdA* strain was transformed with plasmids containing *urdA* from *Aspergillus nidulans* and its *Aspergillus* *flavus* homolog respectively. (A) Linear diagram corresponding to the *A. nidulans urdA* complementation vector. (B) Diagnostic PCR was used to confirm the reintegration of *urdA* in the genome using primers ANurdA-comF-NotI and ANurdA-comR-SpeI (Table S2), labeled in this figure as F and R respectively. The expected 4.4 kb PCR product was obtained. (C) Confirmation of the integration of the *A.flavus urdA* into the genome of the *A. nidulans* Δ*urdA* host strain was carried out using a similar strategy with primers AflurdA-comF-NotI and AflurdA-comR-SpeI (Table S2). The expected 3.7 kb PCR product was obtained.


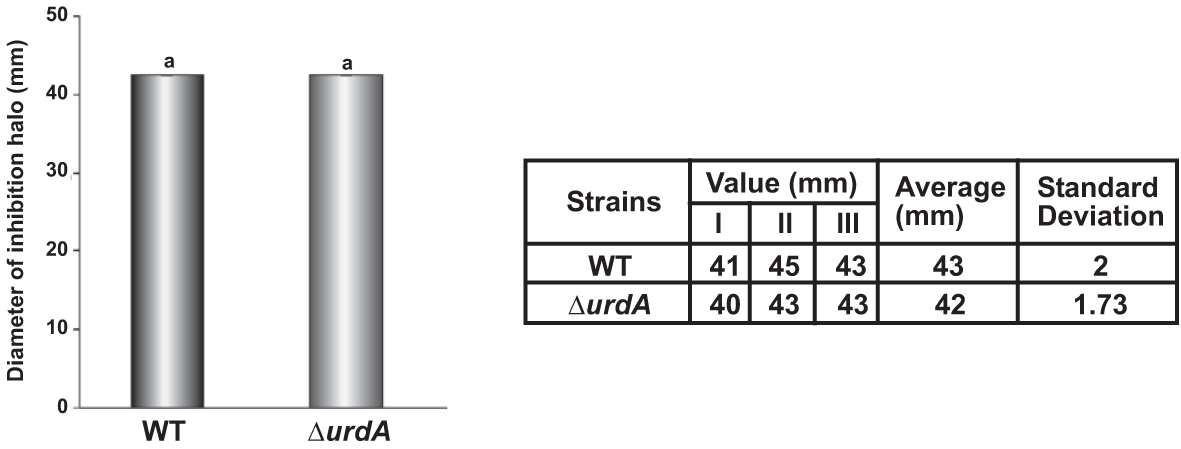


**Figure S5. *urdA* does not affect penicillin (PN) production in *A. nidulans*.** Extracts of wild type (WT) and Δ*urdA* were analyzed for penicillin presence using the bioassay described in Materials and Methods. Diameter of PN growth inhibition halos in mm is shown. Values shown are means of triplicates. Error bars represent standard error.


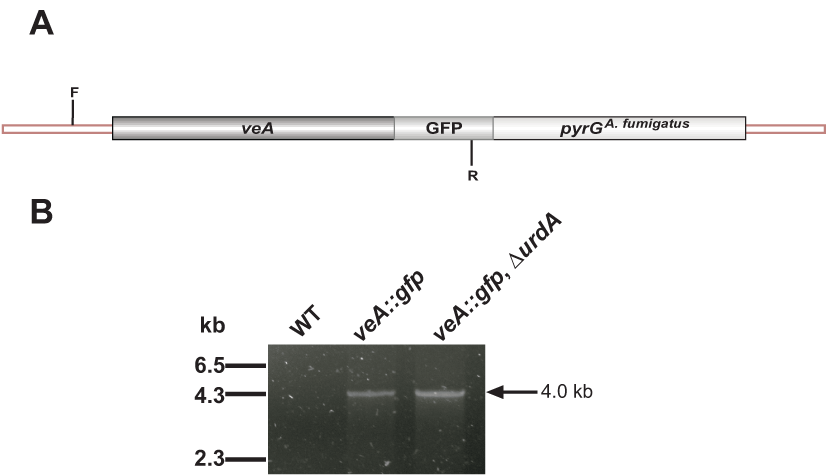


**Figure S6. Generation of the *veA::gfp* and** *veA::gfp* Δ*urdA* **strains.** (A) The *veA::gfp::pyrG^A.fumigatus^* and *veA::gfp::pyrG^A.fumigatus^* Δ*urdA* strains was generated as follows: primers AnidveA_P7 and ANVeASTagP4 (Table S2) were used to PCR amplify a 6.6 kb fragment containing a *veA::gfp::pyrG^A. fumigatus^* fragment from *A. nidulans* T-17 strain (Stinnett et al., 2007). The PCR product was then transformed into *A. nidulans* TSSP7.1 and TSSP4.1 strains (Table S1). (B) Diagnostic PCR was used to confirm the integration of *veA*::*gfp* in the host strains after transformation, using primers VeAFnest and Gfp-mid-R. The expected 4.0 kb PCR product was obtained. Table S1. Fungal strains used in this study. Table S2. Primers used in this study.

References

1. Stinnett, S.M.; Espeso, E.A.; Cobeno, L.; Araujo-Bazan, L.; Calvo, A.M. *Aspergillus* *nidulans* VeA subcellular localization is dependent on the importin alpha carrier and on light. *Mol.* *Microbiol*. **2007**, *63*, 242–255, doi:10.1111/j.1365-2958.2006.05506.x.

© 2018 by the authors. Submitted for possible open access publication under the
terms and conditions of the Creative Commons Attribution (CC BY) license (http://creativecommons.org/licenses/by/4.0/).
