# Supplementary material for: UrdA Controls Secondary Metabolite Production and the Balance between Asexual and Sexual Development in Aspergillus nidulans
Source: Genes (Basel). 2018 Nov 23;9(12):570. doi: 10.3390/genes9120570 (PMC6316066; doi:10.3390/genes9120570)
Supplement: Supplementary file 1 [file genes-09-00570-s001.zip › genes-382948-supplementary-tables.docx]

**Table S1.** Fungal strains used in this study

| **Strain** | **Pertinent genotype** | **Source** |
| --- | --- | --- |
| FGSC4 | Wild type | FGSC* |
| BD834 | *∆urdA::pyrG^A. fum^; pyrG89; pyroA4* | This study |
| TRV50.2 | Wild type | [73] |
| TRV50 | *pyroA4*, *∆nkuA*::*argB*; *veA*+ | [73] |
| T-17 | *veA::gfp:: pyrG^A.fum^ ; pyrG89; pyroA4* | [30] |
| RJMP1.49 | *pyrG89;argB2; ∆nku::argB; pyroA4* | [74] |
| TXF3.1 | *pyrG89;argB2; ∆nku::argB; ∆veA::pyro; pyroA4* | [75] |
| TXFp2.1 | *∆veA::pyrG^A.fum^ ; pyrG89; argB2, ;∆nku::argB; pyroA4,* | [75] |
| TSSP1.1 | *∆urdA::pyrG^A.fum^  ; pyrG89; argB2; ∆nku::argB; pyroA4;* | This study |
| TSSP3.1 | *veA::gfp::pyrG^A.fum^; pyrG89, ∆nku::argB; argB2;* | This study |
| TSSP4.1 | *pyrG89, argB2;, ∆nku::argB; , ∆urdA::pyroA pyroA4* | This study |
| TSSP6.1 | *pyrG89; veA::gfp::pyrG^A.fum^ ; ∆nku::argB; argB2 ; ∆urdA::pyroA ; pyroA4;* | This study |
| TSSP7.1 | *pyrG89; argB2; ∆nkuA::argB* | [75] |
| TSSP13.1 | *pyrG89 ; ∆urdA::pyrG^A.fum^ argB2; ∆nku::argB; ∆veA::pyroA; pyroA4* | This study |
| TSSP23.1 | *pyrG89; argB2; ∆nkuA::argB; alcA::brlA::gpdA::pyroA; pyroA4,* | This study |
| TSSP25.1 | *∆urdA::pyrG ^A. fum^;pyrG89;argB2; ∆nkuA::argB* | This study |
| TSSP26.1 | *∆urdA::pyrG ^A. fum^; pyrG89; ∆nkuA::argB; argB2;; alcA::brlA::gpdA::pyroA; pyroA4* | This study |
| CA14 | *niaD-;pyrG-; Δku70* | USDA** |
| TSSP27.1 | *∆urdA::pyrG^A.fum^ ;pyrG89; ∆nkuA::argB: argB2; urdA*::*pyro; pyroA4* | This Study |
| TSSP28.1 | *∆urdA::pyrG ^A. fum^; pyrG89; ∆nkuA::argB; argB2; urdA^A.flavus^*::*pyroA;pyroA4* | This Study |
| *FGSC, Fungal Genetics Stock Center.  **USDA, United State Department of Agriculture | | |

**Table S2.** Primers used in this study

| **NAME** | **SEQUENCE (5’ → 3’)** |
| --- | --- |
| urdA-P0 | GGATCTCCTGGCGAGTCGTG |
| urdA-P1 | AAAGGCTGAGCAACTGGGAAGC |
| urdA-P2 | AGCCTAAACGCAACCTTGTCTCG |
| urdA-P3 | CGGTTCTCTGCTGTCATCTGGC |
| urdA-P4 | GAAGCGTGTCCTCGTCACTAGAAC |
| urdA-P5-Pyro | CGAGACAAGGTTGCGTTTAGGCTACCCTGGCGTTACCCAACTTA |
| urdA-P6-Pyro | GCCAGATGACAGCAGAGAACCGGGGCGACACGGAAATGTTGAATAC |
| urdA-P7 | AGGTCCTGGATCTCACACTGTCG |
| urdA-P8 | CTTCCACGTCACACCACCCT |
| urdA-P5-pyrG | CGAGACAAGGTTGCGTTTAGGCTACCGGTCGCCTCAAACAATGCTCT |
| urdA-P6-pyrG | GCCAGATGACAGCAGAGAACCGGTCTGAGAGGAGGCACTGATGCG |
| pyroAR | CGCGGAGAAGCTCATAGG |
| Gfp-mid-R | CGTCTTCGATGTTGTGGCGGG |
| pyrG_Afum_F | GATGTGACGACAACCCGAGAACTCC |
| pyrG_Afum_R | GAGCAGCGTAGATGCCTCGACN |
| VeAFnest | CAACGCTCCTGAACGCCCT |
| gpdApromoF | AAGTACTTTGCTACATCCATACTCC |
| ANurdA-comF-NotI | AAAAAAAAAAAGCGGCCGCAGGTCCTGGATCTCACACTGTCG |
| ANurdA-comR-SpeI | AAAAAAACTAGTGACTTCCACGTCACACCACCC |
| AflurdA-comF-NotI | AAAAAAAAAAAGCGGCCGCGGATGCTGGACTCACGATGGTATTCTC |
| AflurdA-comR-SpeI | AAAAAAACTAGTCCAGACAAACACGCTTCGGGATTG |
| AN_alcA(P)_F | GGATCTGCGATGCTCCATAACCG |
| ANbrlA_R | CGTAGTCGGGGCTGTTCTCG |
| veAF5'UTR | TGTACAGCGTGCCGCCATAG |
| veAGFP | CGT ATC CCT CAG GCA TGG CG |
| ANVeASTagP4 | TCGAGAGGCTGAAGCAGAAGACA |
| AnidveA_P7 | GGAATTACCGACTGGGGGAACCGAG |
| pyrG_FGSC4_F | GTCGTTGGACAACCTCTTGGAGC |
| pyrG_FGSC4_R | CCGATGCGATTGTCAAGTGAGTTGG |
| Anidpyro_5UTR_F | GCCGAAAAGGACCACGAATACCCGC |
| Anidpyro_3UTR_R | CACCGCCAACGGAGACAATCAAGCC |
| asp1eQ_18S | GAGACCTCGGCCCTTAAATAGCCCGGTC |
| 18S | CCCAGAACATCTAAGGGCATCACAGACCTGTTATTGC |
| 17352-ANurdA_Q3F | CTCCAGCAGCAGAACGAGCAAC |
| 17353-ANurdA_Q3R | CGATGTGTTGAACCGAGCAGAGTC |
| brlAQF | AGCTGCCTGGTGACGGTAGTTGTTGTTGGTGTTGC |
| brlAQR | CAGGAACGAATGCCTATGCCCGACTTTCTCTCTGGA |
| ANabaA-Q1 | GCACTCACGCAACCTCTTCTTACC |
| ANabaA-Q2 | CGTCTGAGAGTGATGAGCTTAGGTGG |
| ANwetA-Q1 | CGAAGACGATCCGCCATCACCGA |
| ANwetA-Q2 | GGTTAAAGCAGATGTGCCTGTCTGG |
| steAQF | TCCAGCAAATGGAACCGTGGAATCAGGTGCTC |
| steAQR | GAAGGGATGGGGCAAGAATGAGACTTCTGCGGGTAA |
| nsdDQF | CATCTCACCAGCCACAATTACAGGCGGAACCATCAC |
| nsdDQR | TTGCGAGCCAGACACAGAGGTCATAACAGTGCTTGC |
| aflRQ1 | ATGGAGCCCCCAGCGATCAGCCAG |
| aflRQ2 | TTGGTGATGGTGCTGTCTTTGGCTGCTCAAC |
| stcU_Fwd | GTCTCGATGGAAAAGTCGCTCTGGTAACTGGGG |
| stcU_Rev | CATGCCCGAACGAGACAATTCCCGCGTTAG |
| ANnosA-Q1 | GCACAAGCCGTCCTTCAGACC |
| ANnosA-Q2 | CGTATGGGTTCGGAGCGTCC |
